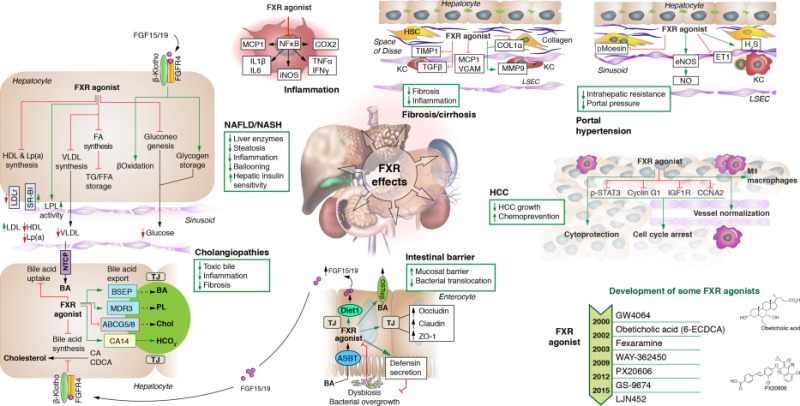# Liver Capsule: FXR agonists against liver disease

**DOI:** 10.1002/hep.28836

**Published:** 2016-10-03

**Authors:** Claudia D. Fuchs, Philipp Schwabi, Thomas Reiberger, Michael Trauner

**Affiliations:** ^1^ Division of Gastroenterology and Hepatology, Department of Internal Medicine IIIMedical University of ViennaViennaAustria

**Abbreviations**
ASBT: apical sodium‐dependent bile acid transporter
BA: bile acid
BSEP: bile salt export pump
CA: cancer antigen
CDCA: chenodeoxycholic acid
Chol: cholesterol
Col1α, collagen 1α; COX2: cyclooxygenase 2
eNOS: endothelial NO synthase
ET1: endothelin 1
FA: fatty acid
FFA: free fatty acid
FGF: fibroblast growth factor
FGFR: FGF receptor
FXR: farnesoid X receptor
HCC: hepatocellular carcinoma
HDL: high‐density lipoprotein
IFN: interferon
IGF1R: insulin‐like growth factor 1 receptor
IL: interleukin
iNOS: inducible NO synthase
KC: Kupffer cell
LDL: low‐density lipoprotein
LDLr: LDL receptor
Lp(a), lipoprotein a; LSEC: liver sinusoidal endothelial cell
MCP1: monocyte chemoattractant protein 1
MDR: multidrug resistance
MMP: matrix metalloproteinase
NAFLD: nonalcoholic fatty liver disease
NASH: nonalcoholic steatohepatitis
NFκB: nuclear factor kappa B
NO: nitric oxide
NTCP: sodium/taurocholate cotransporting polypeptide
PL: phospholipids
SR‐BI: scavenger receptor class B type 1
p‐STAT3: phosphorylated signal transducer and activator of transcription 3
TG: triglyceride
TGF: transforming growth factor
TIMP: tissue inhibitor of metalloproteinase
TJ: tight junction
TNF: tumor necrosis factor
VCAM: vascular cell adhesion molecule
VLDL: very low‐density lipoprotein
ZO‐1: zona occludens 1.